# Efficacy, acceptability, and tolerability of antidepressant treatments for patients with post-stroke depression: a network meta-analysis

**DOI:** 10.1590/1414-431X20187218

**Published:** 2018-05-07

**Authors:** B. Qin, H. Chen, W. Gao, L.B. Zhao, M.J. Zhao, H.X. Qin, W. Chen, L. Chen, M.X. Yang

**Affiliations:** 1 Department of Neurology, Affiliated Liuzhou People's Hospital, Guangxi University of Science and Technology, Liuzhou, Guangxi, China; 2 Department of Neurology, Yongchuan Hospital, Chongqing Medical University, Chongqing, China; 3 Department of Pharmacy, The Second Affiliated Hospital, Xinxiang Medical University, Xinxiang, China; 4 Department of Pharmacy, Henan Mental Hospital, Xinxiang, China

**Keywords:** Antidepressants, Post-stroke depression, Randomized controlled trials, Network meta-analysis

## Abstract

The aim of this study was to investigate the efficacy, acceptability, and tolerability of antidepressants in treating post-stroke depression (PSD) by performing a network meta-analysis of randomized controlled trials of the current literature. Eligible studies were retrieved from online databases, and relevant data were extracted. The primary outcome was efficacy as measured by the mean change in overall depressive symptoms. Secondary outcomes included discontinued treatment for any reason and specifically due to adverse events. Fourteen trials were eligible, which included 949 participants and 9 antidepressant treatments. Few significant differences were found for all outcomes. For the primary outcome, doxepin, paroxetine, and nortriptyline were significantly more effective than a placebo [standardized mean differences: −1.93 (95%CI=−3.56 to −0.29), −1.39 (95%CI=−2.59 to −0.21), and −1.25 (95%CI=−2.46 to −0.04), respectively]. Insufficient evidence exists to select a preferred antidepressant for treating patients with post-stroke depression, and our study provides little evidence that paroxetine may be the potential choice when starting treatment for PSD. Future studies with paroxetine and larger sample sizes, multiple medical centers, and sufficient intervention durations is needed for improving the current evidence.

## Introduction

Depression is the most common neuropsychiatric complication experienced after stroke, and it has a significant negative impact on patients' rehabilitation, functional recovery, quality of life, and even survival rate (1). Previous critical appraisals of the literature have reported that 20 to 50% of all post-stroke patients suffer from depression (2,3). The proper management of post-stroke depression (PSD) is critical to reduce morbidity and mortality. Among the different modalities for treating this population, the use of antidepressants is supported by relatively sufficient evidence from multiple studies (4–6). In these studies, PSD was categorized on the basis of psychiatric interviews using standard diagnostic criteria, such as the Diagnostic and Statistical Manual of Mental Disorders (e.g., DSM-III-R, DSM-IV), or another validated rating scale for depression, such as the Montgomery Åsberg Depression Rating Scale (MADRS). However, cognitive, language, and functional impairments in patients who have suffered an acute stroke may result in difficulties in recognizing PSD, resulting in the under-diagnosis and under-treatment of this complication. There is a consensus that if PSD is left untreated, it may have a negative effect on the patient's functional recovery. A previous study showed that major and minor cases of depression can result in disability, failure to return to work, impaired interpersonal functioning, and mortality (7). Therefore, PSD has a negative effect on the functional recovery of stroke patients.

To date, antidepressants such as tricyclic antidepressants (TCAs), monoamine oxidase inhibitors (MAOIs), serotonin-norepinephrine reuptake inhibitors (SNRIs), and selective serotonin reuptake inhibitors (SSRIs) have been used to treat PSD. There are no guidelines for treating PSD, and the effectiveness of interventions is not well established. In a previous Cochrane review, Hackett et al. found a small but significant effect of antidepressants in treating depression, with a significant increase in adverse events (8). According to the meta-analysis conducted by Price et al. (9), the use of antidepressants may be indicated for both major and minor depressive disorders, but there are no specific guidelines for the selection of drugs. A recent meta-analysis suggests that antidepressant treatment confers potentially positive effects in patients with PSD compared with a placebo treatment (10).

However, these reviews reported only the effect of antidepressants on PSD compared with a placebo and therefore are of restricted use for clinical practice. A few systematic reviews have looked at the comparative effectiveness of different antidepressants, but they considered only direct evidence and did not address different drug preparations (8,11). In the Network meta-analysis (NMA), all interventions that have been tested in randomized controlled trials (RCTs) can be simultaneously compared, and their effects can be estimated relative to each other and to a common reference condition (e.g., placebo). Therefore, NMA allows an integrated analysis of all RCTs that compare different antidepressants, e.g., SSRIs, SNRIs, and TCAs, either with each other or with a placebo treatment, while fully respecting the randomized nature of the studies (12). Our previous study showed that duloxetine has a potential beneficial effect for depression in young depressive populations (13). Moreover, our recent NMA of antidepressant use in treating depressive disorders in children and adolescents suggests that only fluoxetine reduces the severity depression symptoms (14). In the present study, we assessed the effectiveness, acceptability, and tolerability of different preparations of antidepressants in treating PSD by integrating all available direct and indirect evidence in an NMA.

## Material and Methods

### Data sources and search strategy

We conducted a systematic search of the PubMed, EMBASE, Cochrane Central Register of Controlled Trials, Web of Science, PsycINFO, World Health Organization International Trial Registry, and clinicaltrials.gov databases from their inception to March 2017 using search terms such as “post-stroke depression” (see Supplementary Tables S1–S5). Only studies published in English were included in this investigation. Moreover, we inspected the reference lists of the included studies and previous reviews of the use of antidepressants in treating PSD. Additionally, we reviewed all the references listed in the trials we found, and investigators were also contacted via telephone or email about unpublished trials.

### Selection criteria

Studies were included if they involved a RCT assessing any antidepressant available worldwide, at any dose and administered in any form, that were compared with other antidepressants or a placebo for treating PSD, and if the antidepressants were used as a monotherapy. The study subjects met the following criteria: 1) no limitations on gender, age, race, region, or nationality of the patients; 2) patients were diagnosed as having had a stroke clinically and/or by computed tomography or nuclear magnetic resonance imaging, and 3) patients had a diagnosis of depression, as confirmed on the basis of DSM criteria or other validated rating scales for depression. Exclusion criteria were as follows: 1) combination therapy, such as an antidepressant combined with psychotherapy, and 2) relevant outcome indexes not reported. Two reviewers independently assessed all citations and discarded those that were irrelevant based on the title of the publication and its abstract. If the article was possibly relevant, we retrieved the full-length article for further assessment. Two reviewers independently selected the trials for inclusion from the rejected citation list. All disagreements were resolved through discussion or following arbitration by a third reviewer, if necessary.

### Outcome measures

The primary outcome was the mean change in overall depressive symptoms, which was assessed in the first instance by a change in depression rating scale scores (difference in scores from baseline to endpoint). When a trial reported multiple scores, the Hamilton Depression Scale (HAMD) was preferred. A negative value indicated greater relief from depressive symptoms. Intention-to-treat datasets were used whenever available. Secondary outcomes were the proportion of patients who discontinued treatment for any reason (acceptability) and the proportion of patients who discontinued treatment due to adverse effects (tolerability). Because an NMA requires reasonable homogeneity, we focused on acute treatments, which we defined as those lasting 8 weeks. If data over 8 weeks were not available, we used data from between weeks 4 and 12 (the data points closest to 8 weeks were given preference).

### Data extraction and quality assessment

Data extraction was performed independently by two reviewers and any discrepancies were resolved via discussion. Extracted data included the study methodology, identification of outcome measures, results, and final conclusions. We also used the risk of bias assessment tool from the Cochrane Handbook to assess the methodological quality of the studies. Assessed quality criteria were randomization, concealed allocation, blinding, incomplete outcome data, selective outcome reporting, and ‘other issues’.

### Statistical analysis

NMA combines direct and indirect evidence for all relative treatment effects and provides estimates with maximum power (15,16). NMA also accounts for the correlation between trial-specific effects and random effects of trials with more than two arms (17). In addition, we estimated the outcomes using Markov chain Monte Carlo analysis implemented in WinBUGS, version 1.4.3 (MRC Biostatistics Unit, UK). To rank the treatments, we used the surface under the cumulative ranking (SUCRA) probabilities (16). The first 30,000 iterations were discarded, and 50,000 additional iterations were then run. Two chains with different initial values were run simultaneously to assess convergence using Gelman-Rubin diagnostic trace plots. Network consistency was then statistically assessed to calculate the differences between direct and indirect estimates in all closed loops in the network using STATA, version 13.0 (STATA Corporation, USA) (18). Moreover, to assess the evidence of inconsistency in the entire network, we used the design-by-treatment model (19), which enabled us to examine the presence of loop and design inconsistencies. Deviance information criterion (DIC) and residual deviance (Dres) parameters were computed to measure model fit (20). For continuous outcomes, relative effect sizes were calculated as standardized mean differences (SMDs). For binary outcomes, relative effect sizes were calculated as odds ratios (ORs). Both types of effect sizes are reported with their 95%CI. Main effects are reported for the effects of drugs compared with those of a placebo, which was chosen as the reference treatment a priori. Sensitivity analysis was performed to explore the impact of double-blinding. The funnel plot was used to identify the possible publication bias if the number of studies was larger than 10.

## Results

**Figure 1.**
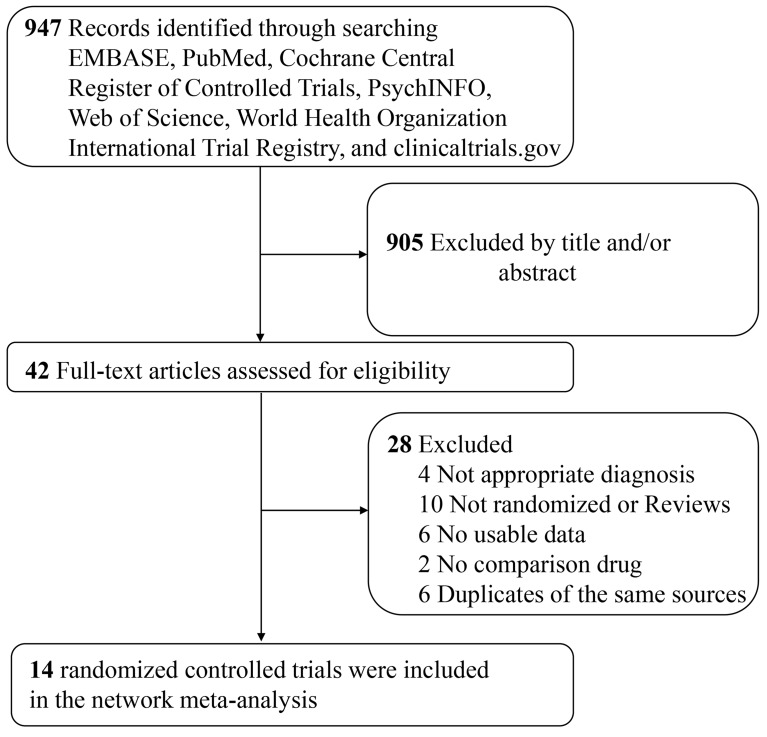
Search and study selection process (PRISMA diagram).

According to the inclusion criteria described above, 14 studies, reported between 1984 and 2012 and involving 949 patients, were included in our NMA (21–34). A flow chart (Figure 1) shows the study selection details. Table 1 lists the included studies and summarizes their characteristics. The mean age of participants was 60.0 years, and 46% of participants for whom data were reported were women.

**Table 1.** Description of included studies.

Study	Interventions (n)	Mean age	Female (%)	Depression criteria	Duration	Depression rating scale
Andersen 1994 (21)	Citalopram (33); Placebo (33)	67.0	60.6%	DSM-III-R; HAMD (17 items) >12	6 weeks	HAMD (17 items)
Chen 2002 (22)	Doxepin (24); Paroxetine (24); Placebo (24)	not stated	not stated	Classification and diagnostic criteria of psychosis in China	8 weeks	HAMD (17 items)
Fruehwald 2003 (23)	Fluoxetine (28); Placebo (26)	64.4	42.0%	Psychiatric interview, HAMD (17 items) >15	12 weeks	HAMD (17 items)
Karaiskos 2012 (24)	Citalopram (20); Duloxetine (20); Sertraline (20)	52.6	not stated	DSM-IV	12 weeks	HAMD
Li 2008 (25)	Fluoxetine (60); Placebo (30)	68.7	53.3%	HAMD (17 items) >20	8 weeks	HAMD (17 items)
Lipsey 1984 (26)	Nortriptyline (17); Placebo (22)	60.9	35.3%	DSM-III	6 weeks	HAMD (17 items)
Murray 2005 (27)	Sertraline (62); Placebo (61)	70.7	52.0%	DSM-IV, MADRS ≥10	6 weeks	MADRS
Ponzio 2001 (28)	Paroxetine (112); Placebo (117)	65.0	45.4%	MADRS ≥18	8 weeks	MADRS
Raffaele 1996 (29)	Trazodone (11); Placebo (11)	70.0	40.9%	DSM-III-R	45 days	ZDS
Reding 1986 (30)	Trazodone (11); Placebo (6)	68.0	29.4%	DSM-III	32 days	ZDS
Robinson 2000 (31)	Fluoxetine (23); Nortriptyline (16); Placebo (17)	62.7	44.6%	DSM-IV, HAMD (28 items) ≥12	12 weeks	HAMD (28 items)
Tzavellas 2010 (32)	Duloxetine (15); Sertraline (15)	58.6	30.0%	not stated	12 weeks	HAMD (21 items)
Wiart 2000 (33)	Fluoxetine (16); Placebo (15)	67.6	51.6%	ICD-10	45 days	MADRS
Ye 2006 (34)	Paroxetine (30); Imipramine (30)	57.5	33.3%	HAMD (24 items) >21	12 weeks	HAMD (24 items)

DSM-III/-R: Diagnostic and Statistical Manual of Mental Disorders, 3rd edn., revised; DSM-IV: Diagnostic and Statistical Manual of Mental Disorders; HAMD: Hamilton Depression Scale; ICD: International Classification of Diseases; MADRS: Montgomery Asberg Depression Rating Scale; ZDS: Zung Depression Scale.

Participants were assigned to a placebo group or to one of the following 9 treatment interventions: citalopram, doxepin, duloxetine, fluoxetine, imipramine, nortriptyline, paroxetine, sertraline, and trazodone. Four open-label randomized trials were included (22,24,32,34). The mean treatment duration was 8.4 weeks (SD 2.7; range 5–12). We assessed all included trials for a risk of bias. Sequence generation and allocation concealment were adequately described in nine trials. Other risks of bias of the included studies are presented in Supplementary Figures S1 and S2.

**Figure 2.**
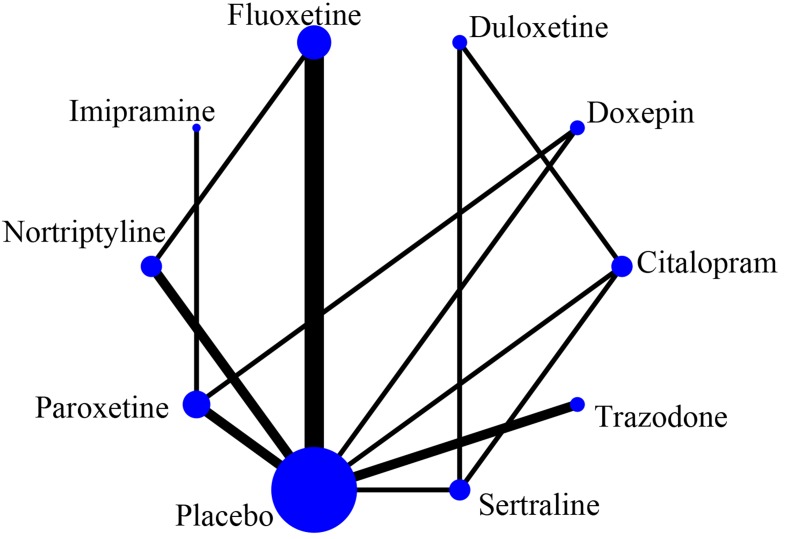
Network plot for mean overall change in depressive symptoms. The size of the nodes corresponds to the number of trials that studied the treatment. Directly comparable treatments are linked with a line; the thickness of the line corresponds to the number of trials that assessed the comparison.

Figure 2 shows the network of eligible comparisons for the NMA. Of 45 possible pair-wise comparisons among 10 interventions, 13 direct comparisons were made for our primary outcome: efficacy (mean change in overall depressive symptoms, the networks for each outcome are provided in Supplementary Figure S3).

### Primary outcome

**Figure 3.**
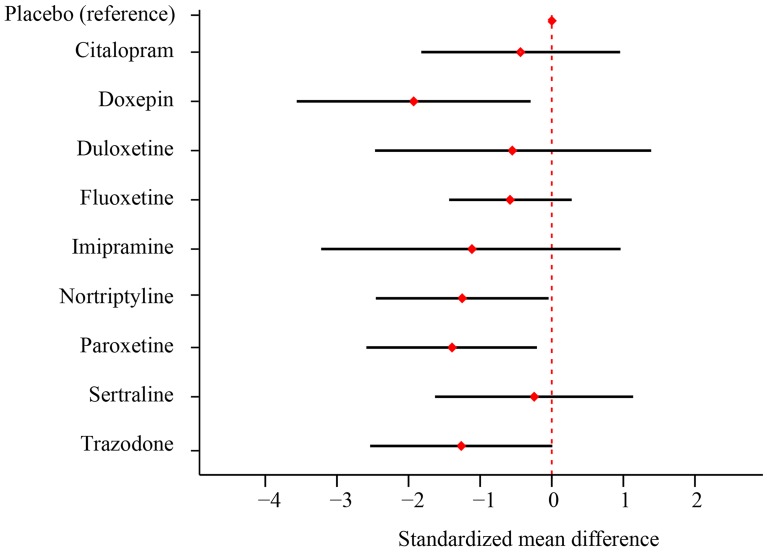
Forest plot for mean overall change in depressive symptoms of antidepressants compared with placebo. Data are the standardized mean difference and 95% confidence intervals compared to placebo.

The results of the NMA for the primary outcome (efficacy) are summarized in Table 2. In addition, Figure 3 presents forest plots of SMDs for antidepressants included in the closed-loop network in comparison with a placebo. SUCRA values are given in Supplementary Figures S4 and S5. The heterogeneity of variance of the random-effects NMA models for primary outcomes was 0.82. Cohen’s rule standardized a small SMD as −0.20, medium as −0.50, and large as −0.80. Few statistically significant differences were found. Doxepin, paroxetine, and nortriptyline were significantly more effective than a placebo [SMDs=−1.93 (95%CI=−3.56 to −0.29), −1.39 (95%CI=−2.59 to −0.21), and −1.25 (95%CI=−2.46 to −0.04), respectively], but they did not show a significant difference compared with any other antidepressant. In terms of rankings, doxepin was ranked first, followed by paroxetine, nortriptyline, and trazodone, but the results for trazodone did not show a significant difference compared with any other antidepressant or a placebo.

**Table 2.** Primary outcome of mean change in overall depressive symptoms.

Doxepin									
−0.54 (−2.17 to 1.10)	Paroxetine								
−0.68 (−2.71 to 1.35)	−0.14 (−1.86 to 1.55)	Nortriptyline							
−0.66 (−2.74 to 1.41)	−0.13 (−1.89 to 1.61)	0.01 (−1.75 to 1.76)	Trazodone						
−0.81 (−3.19 to 1.57)	−0.28 (−2.00 to 1.45)	−0.14 (−2.54 to 2.30)	−0.15 (−2.58 to 2.31)	Imipramine					
−1.38 (−3.91 to 1.14)	−0.84 (−3.13 to 1.41)	−0.70 (−2.96 to 1.56)	−0.71 (−3.03 to 1.60)	−0.56 (−3.44 to 2.27)	Duloxetine				
−1.35 (−3.20 to 0.50)	−0.81 (−2.29 to 0.65)	−0.67 (−2.01 to 0.66)	−0.68 (−2.22 to 0.85)	−0.53 (−2.81 to 1.72)	0.03 (−2.08 to 2.14)	Fluoxetine			
−1.49 (−3.64 to 0.65)	−0.96 (−2.80 to 0.87)	−0.81 (−2.66 to 1.03)	−0.83 (−2.72 to 1.05)	−0.68 (−3.21 to 1.82)	−0.11 (−1.79 to 1.56)	−0.15 (−1.77 to 1.49)	Citalopram		
−1.68 (−3.83 to 0.46)	−1.15 (−2.98 to 0.68)	−1.01 (−2.84 to 0.82)	−1.02 (−2.89 to 0.87)	−0.87 (−3.40 to 1.63)	−0.31 (−1.98 to 1.37)	−0.34 (−1.95 to 1.28)	−0.19 (−1.59 to 1.22)	Sertraline	
**−1.93 (−3.56 to −0.29)**	**−1.39 (−2.59 to −0.21)**	**−1.25 (−2.46 to −0.04)**	−1.26 (−2.54 to 0.01)	−1.12 (−3.22 to 0.96)	−0.55 (−2.47 to 1.39)	−0.58 (−1.43 to 0.28)	−0.44 (−1.82 to 0.95)	−0.25 (−1.63 to 1.14)	Placebo

Drugs are reported in order of efficacy ranking, and outcomes are standardized mean differences (SMDs; 95% confidence intervals). SMDs of less than 0 indicate that the treatment specified in the column is more efficacious than placebo. Bold results indicate statistical significance (P<0.05, network meta-analysis).

### Acceptability

Discontinuing treatment for any reason was used as a measure of acceptability and Table 3 reports ORs and 95%CI based on this measure. SUCRA values are reported in Supplementary Figures S6 and S7. The heterogeneity of variance of the random-effects NMA models for all-cause discontinuation was 0.42. Few statistically significant differences were found. Only paroxetine was more acceptable than doxepin [OR=0.11 (95%CI=0.03 to 0.85)], but paroxetine did not show a significant difference compared with a placebo.

**Table 3.** Secondary outcome of treatment discontinuation due to any reason.

Paroxetine									
0.57 (0.15 to 4.12)	Nortriptyline								
0.81 (0.21 to 1.87)	1.02 (0.24 to 2.83)	Placebo							
2.38 (0.03 to 14.39)	3.12 (0.04 to 18.51)	0.32 (0.06 to 16.91)	Trazodone						
0.32 (0.09 to 1.87)	0.41 (0.12 to 2.11)	0.54 (0.21 to 1.84)	0.18 (0.03 to 12.28)	Fluoxetine					
0.04 (0.01 to 27.26)	0.05 (0.01 to 33.47)	0.07 (0.01 to 34.15)	0.02 (0.00 to 64.35)	0.10 (0.02 to 58.07)	Duloxetine				
0.46 (0.05 to 1.63)	0.58 (0.06 to 2.32)	0.34 (0.11 to 1.74)	0.11 (0.02 to 10.03)	1.05 (0.12 to 4.04)	6.20 (0.01 to 39.79)	Sertraline			
0.03 (0.01 to 2.94)	0.03 (0.01 to 6.15)	0.04 (0.01 to 5.59)	0.02 (0.00 to 16.57)	1.77 (0.01 to 11.07)	14.11 (0.00 to 79.58)	0.07 (0.01 to 17.71)	Imipramine		
0.09 (0.02 to 1.23)	0.10 (0.02 to 1.69)	0.15 (0.04 to 1.38)	0.05 (0.01 to 6.47)	0.21 (0.04 to 2.96)	0.03 (0.01 to 18.81)	0.30 (0.06 to 4.25)	0.16 (0.02 to 43.48)	Citalopram	
**0.11 (0.03 to 0.85)**	0.12 (0.03 to 1.48)	0.18 (0.05 to 1.18)	0.06 (0.01 to 6.20)	0.24 (0.06 to 2.68)	0.02 (0.01 to 30.86)	0.31 (0.07 to 4.60)	0.20 (0.03 to 37.01)	2.23 (0.10 to 12.18)	Doxepin

Drugs are reported in order of efficacy ranking, and outcomes are standardized odds ratios (ORs; 95% confidence intervals). ORs of less than 1 indicate that the treatment specified in the column is better than placebo. Bold results indicate statistical significance (P<0.05, network meta-analysis).

### Tolerability

Discontinuing treatment due to adverse effects was used as a measure of tolerability and Table 4 provides ORs and 95%CI based on this measure. SUCRA values are reported in Supplementary Figures S8 and S9. The heterogeneity of variance of the random-effects NMA models for discontinuation due to adverse effects was 0.42. Paroxetine and placebo were more tolerable than doxepin [OR=0.01 (95%CI=0.00 to 0.54) and 0.01 (95%CI=0.00 to 0.50), respectively].

**Table 4.** Secondary outcome of treatment discontinuation due to adverse effects.

Placebo									
1.36 (0.25 to 4.33)	Paroxetine								
0.38 (0.07 to 19.16)	0.22 (0.04 to 19.86)	Trazodone							
0.28 (0.07 to 2.11)	1.24 (0.26 to 21.86)	0.08 (0.01 to 9.56)	Sertraline						
0.06 (0.01 to 35.10)	0.05 (0.01 to 44.80)	0.02 (0.00 to 63.25)	0.15 (0.03 to 79.49)	Duloxetine					
0.28 (0.08 to 1.81)	0.22 (0.05 to 3.31)	0.09 (0.01 to 8.78)	0.48 (0.10 to 9.82)	9.10 (0.01 to 54.78)	Nortriptyline				
0.24 (0.07 to 1.54)	0.19 (0.04 to 2.83)	0.08 (0.01 to 7.33)	0.41 (0.08 to 8.29)	7.32 (0.01 to 48.93)	0.56 (0.14 to 5.11)	Fluoxetine			
0.12 (0.02 to 2.46)	0.10 (0.02 to 4.01)	0.03 (0.01 to 9.00)	0.27 (0.05 to 7.86)	48.77 (0.04 to 106.90)	0.23 (0.04 to 10.76)	3.92 (0.08 to 23.67)	Citalopram		
0.00 (0.00 to 2.40)	0.00 (0.00 to 1.66)	0.00 (0.00 to 6.64)	0.00 (0.00 to 9.36)	7.06 (0.00 to 36.80)	0.01 (0.00 to 8.87)	1.68 (0.00 to 10.75)	3.20 (0.00 to 20.74)	Imipramine	
**0.01 (0.00 to 0.50)**	**0.01 (0.00 to 0.54)**	0.00 (0.00 to 2.00)	0.02 (0.01 to 2.35)	0.00 (0.00 to 11.44)	0.02 (0.01 to 2.29)	0.02 (0.01 to 2.81)	1.10 (0.18 to 145.33)	0.05 (0.01 to 57.94)	Doxepin

Drugs are reported in order of efficacy ranking, and outcomes are standardized odds ratios (ORs; 95% confidence intervals). ORs of less than 1 indicate that the treatment specified in the column is better. Bold results indicate statistical significance (P<0.05, network meta-analysis).

### Sensitivity analysis

Sensitivity analysis, excluding the four studies without a double-blind design (22,24,32,34,), showed that only nortriptyline was significantly more effective than a placebo [SMD=−1.25 (95%CI=−2.40 to −0.10)] (see Supplementary Table S6).

### Publication bias

Publication bias or small-study effects were explored with a funnel plot technique expanded to the NMA. According to the funnel plot, the observed asymmetry was primarily caused by one small study, suggesting a small-study effect rather than publication bias (Supplementary Figure S10).

### Consistency checking

We checked for consistency in the three treatment loops for each of the primary and secondary outcomes. Consistency was found in one of four comparison loops for the mean change in overall depressive symptoms, zero of four for discontinuation for any reason and zero of four for discontinuation due to adverse effects (for details of the assessments of consistency, see Supplementary Figures S11–S13). The test of entire-network inconsistency showed a significant difference between the consistency and inconsistency models for mean change in overall depressive symptoms (P=0.01) but not for discontinuation for any reason (P=0.46) or discontinuation due to adverse effects (P=0.66; see Supplementary Table S7). However, model fit, as assessed by the consistency parameters DIC and Dres, was good for all of the networks examined (see Supplementary Table S8).

## Discussion

This study performed a comprehensive comparison of the efficacy, tolerability, and acceptability of antidepressants using an NMA. Interventions were grouped into placebo, SSRIs (citalopram, fluoxetine, paroxetine, sertraline), TCAs (doxepin, imipramine, nortriptyline), SNRIs (duloxetine), and trazodone. The efficacy outcome was measured as the mean change in overall depressive symptoms, which was assessed as the change in depression rating scale scores (difference in scores from baseline to endpoint). To assess acceptability and tolerability, we examined the proportions of patients who discontinued treatment for any reason and who discontinued treatment due to adverse effects; a high treatment discontinuation rate indicates low efficacy, concerns regarding safety or the risk to become tolerant to the treatment. To our knowledge, this is a pivotal study to thoroughly explore the efficacy, tolerability and acceptability rankings of antidepressants for treating PSD and include a wide range of outcomes. Doxepin, paroxetine, and nortriptyline were found to be more effective than a placebo. Paroxetine was found to be more acceptable than doxepin. Doxepin was not found to be more tolerable than paroxetine or a placebo. These results indicate that one of the most efficacious treatments (doxepin) might not be the best choice in terms of overall acceptability and tolerability. Moreover, the evidence for nortriptyline was only from trials with small sample sizes, which might result in an exaggerated treatment effect (35).

The most important clinical implication of the results presented here is that paroxetine might be the potential choice when starting treatment for PSD because it appears to have a good balance between efficacy, acceptability, and tolerability. Paroxetine's potential was originally demonstrated in a pivotal study in which it effectively improved the depressive symptoms of patients with PSD (36). In addition, it was also safe and well tolerated. Owing to methodological limitations, such as non-placebo-controlled and open-label designs, the results of this study are not definitive. Our findings are consistent with data from a previous study, and they strengthen the evidence that paroxetine might be the appropriate choice for treating PSD. However, the wide confidence interval of the effect sizes between paroxetine and placebo raises the question of whether this estimate is robust enough to inform clinical practice. Furthermore, in comparison with other antidepressants, paroxetine did not show a significant difference in efficacy outcomes, and in terms of acceptability and tolerability, paroxetine was not better tolerated than placebo. Finally, in the sensitivity analysis, excluding trials without a double-blind design, paroxetine was not significantly more effective than a placebo. The open-label designs might have introduced a bias because patients or investigators might have taken/prescribed concomitant treatments to enhance efficacy based on their knowledge and beliefs of treatment allocation. However, it has been suggested that potential benefits of an open-label design may be sometimes intentionally directed by the need to mimic a daily clinical routine where therapeutic flexibility is needed. Thus, our results should be interpreted and translated into clinical practice with caution due to the uncertain evidence in the present meta-analysis.

A key requirement of pharmacotherapy is achieving a therapeutic dose of the medication for an adequate period of time. The guidelines of the American College of Physicians suggest that antidepressants should be continued for at least 4 months beyond initial recovery and that treatment should be changed if no response has been shown by 6 weeks. Unfortunately, due to the absence of reliable data on an adequate length of time to show a maximal or sustained response for many included studies, it was not possible to assess the long-term effects of antidepressant therapy or to provide information on the most appropriate treatment duration or dose. Therefore, the findings from this analysis for paroxetine apply only to the acute phase (8–12 weeks) of treating depression. Clinicians need to know whether (and to what extent) treatments work within a clinically reasonable period. Clinically, the assessment of efficacy after 12 weeks or more of treatment might lead to large differences in treatment outcomes.

Owing to several limitations, our NMA is not definitive. First, adequate information about randomization and allocation concealment was not reported in most included trials, and this might undermine the validity of our overall findings. Nonetheless, all trials included in this meta-analysis were very similar in terms of design and methodology, and the limited information in terms of quality assessment could be more an issue of what was reported in the text rather than real study design problems, as has been commonly found in other systematic reviews (37). Additionally, we included a broad range of antidepressant studies with varied durations, and with potential unknown differences among participants in different trials. However, we did not identify any systematic differences in participant demographics or initial symptom severity. Second, in our NMA, we found an inconsistency in efficacy outcomes, which was mainly determined by the doxepin-paroxetine-placebo loop, but we found no inconsistencies for acceptability and tolerability outcomes, probably because the discontinuation rate is a more definitive outcome than efficacy, which is measured on a subjective rating scale. We believe that this inconsistency might be a result of a risk of bias involving the open-label designs of two included studies. Some evidence suggests that open-label designs for psychopharmacological clinical trials might enhance efficacy or improve tolerability based on patients' or investigators' knowledge and beliefs regarding treatment allocation (38). It has been suggested that the potential benefits of an unblinded design may outweigh those of a blinded design for psychopharmacological studies (39), and the choice to not blind a study could sometimes be intentional due to the need to mimic a daily clinical routine, where therapeutic flexibility is needed (40). Furthermore, to reduce heterogeneity and inconsistency among trials in the NMA, we excluded studies in which participants displayed “retarded” PSD, which represents a proportion of patients seen in real-world clinical settings. However, we have to acknowledge that this restricts the external validity of our results. Finally, too few studies were included to be able to perform an NMA that addressed the clinically important issue of antidepressant therapy for improving the ability to perform activities of daily living of those with PSD. However, the result of a multicenter clinical trial suggests that paroxetine can effectively improve the quality of life of patients with PSD. In addition, the data from this multicenter clinical trial support, in principle, the use of paroxetine as a preferred treatment option for improving measures related to activities of daily living.

Our analysis suggests that paroxetine may be a potential treatment option for PSD patients, however, the extent to which symptom reduction was clinically meaningful is still uncertain. A larger sample size, which has some disadvantages, might be required for an ideal trial. The increased real-world applicability of the results would, in our opinion, offset the disadvantages.

## Supplementary Material

Click here to view [pdf]
